# Diabetes through a 3D lens: organoid models

**DOI:** 10.1007/s00125-020-05126-3

**Published:** 2020-03-27

**Authors:** Anastasia Tsakmaki, Patricia Fonseca Pedro, Gavin A. Bewick

**Affiliations:** Faculty of Life Sciences and Medicine, School of Life Course Sciences, Department of Diabetes, Diabetes Research Group, Hodgkin Building, King’s College London, Guy’s Campus, London, SE1 1UL UK

**Keywords:** 3D culture, Beta cells, Diabetes, Diabetic complications, Disease modelling, Glucose homeostasis, Obesity, Organoids, Review, Stem cells

## Abstract

Diabetes is one of the most challenging health concerns facing society. Available drugs treat the symptoms but there is no cure. This presents an urgent need to better understand human diabetes in order to develop improved treatments or target remission. New disease models need to be developed that more accurately describe the pathology of diabetes. Organoid technology provides an opportunity to fill this knowledge gap. Organoids are 3D structures, established from pluripotent stem cells or adult stem/progenitor cells, that recapitulate key aspects of the in vivo tissues they mimic. In this review we briefly introduce organoids and their benefits; we focus on organoids generated from tissues important for glucose homeostasis and tissues associated with diabetic complications. We hope this review serves as a touchstone to demonstrate how organoid technology extends the research toolbox and can deliver a step change of discovery in the field of diabetes.

**Electronic supplementary material:**

The online version of this article (10.1007/s00125-020-05126-3) contains a slideset of the figures for download, which is available to authorised users.

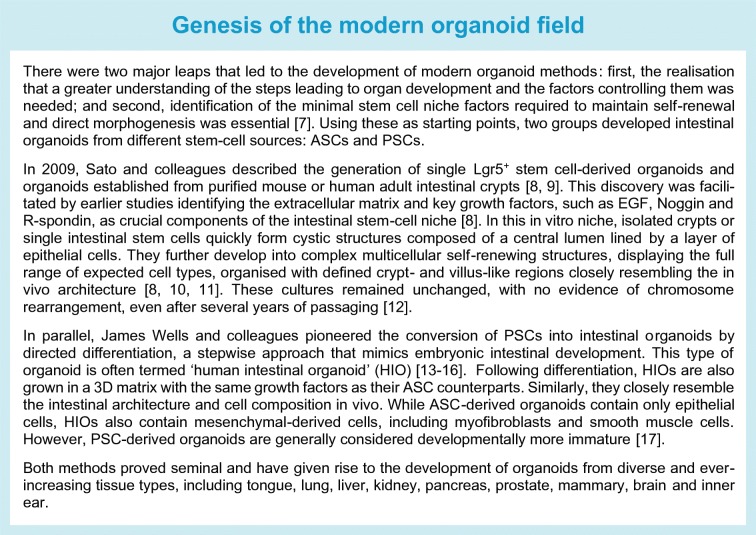

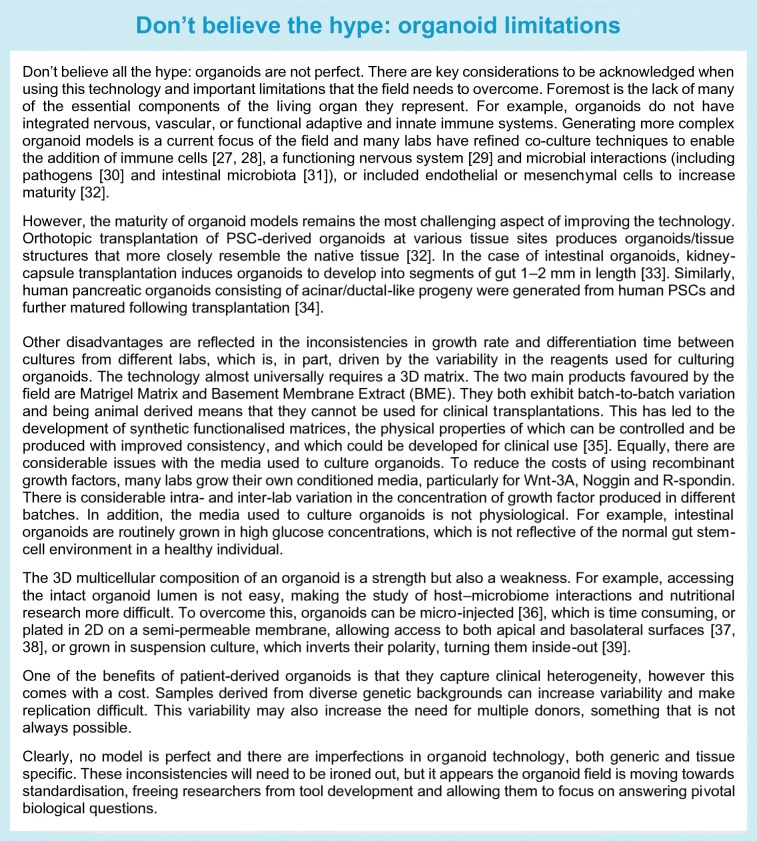





## What are organoids?

The past decade has born witness to the meteoric rise of organoid technology, a long-term heterogenous stem cell-based culture technique, chosen as *Nature* method of the year 2017 [1]. Organoids have transformative potential; they will help us to better model human diseases, leading to the development of novel treatments, the revolutionising of personalised medicine and the acceleration of regenerative medicine. But what exactly are organoids? The definition of an organoid has been nebulous, owing to its use in describing the many different types of 3D culture systems developed in the last 50 years [2]. Since the development of intestinal stem cell-derived organoid cultures in 2009 (discussed in the Text box: ‘Genesis of the modern organoid field’), the term now refers to a specific set of working criteria. The current definition requires an organoid to be established from pluripotent stem cells or adult stem/progenitor cells, to demonstrate a 3D structure resembling the in vivo organ landscape, exhibit an array of cell types found in vivo and demonstrate some aspects of the specialised functions of the tissues [3–5].

## A step change in disease modelling

A staple tool of the medical research community is the modelling of cellular function and disease in vitro. In a dish, experimental variables can be accurately controlled, cells can easily be manipulated, and outputs measured by standard and high-throughput technologies. Cell lines and explant cultures have traditionally been used, but both have limitations. 2D-cultured cells may not act in vitro as they would in vivo*,* because they are not grown in conditions that adequately mimic the in vivo microenvironment. In addition, cell lines are transformed, making these cells far from ‘normal’, calling into question how representative of physiology they really are. In contrast, explant cultures are a more physiologically relevant model as they contain a heterogenous population of primary cells representative of the tissue of origin. However, these cultures cannot be maintained for long periods, which increases the need for multiple tissue donors, and they are generally difficult to genetically manipulate. Organoid cultures address many of these shortcomings as they are complex 3D, multicellular, self-renewing primary tissue structures that can be cultured over months to years without losing their faithful near physiological representation of the tissues they mimic [6]. These properties provide the research community with a step change in our ability to model diseases on the bench.

## What can organoids do for you?

**Fig. 1:**
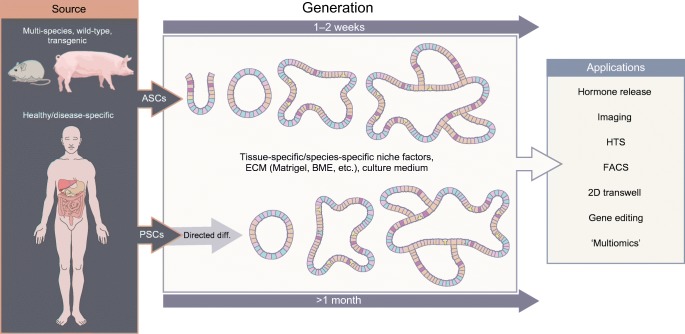
Organoid generation and applications. Organoids can be generated from multiple species and multiple tissues. There are two general sources: adult tissue-resident stem cells (ASCs) and pluripotent stem cells (PSCs). Generation of PSC-derived organoids requires directed differentiation towards the tissue of interest, whereas those derived from ASCs do not. Both sources require specific niche factors and an extracellular matrix (ECM) in which they form 3D multicellular organoids mimicking the tissue of interest. The schematic shows archetypal intestinal organoid development; the different colours indicate different cell types. Both types of organoid can be utilised in many downstream methods, as shown (this list is not exhaustive). BME, Basement Membrane Extract; diff., differentiation; FACS, fluorescence-activated cell sorting; HTS, high-throughput screening. This figure is available as part of a downloadable slideset

The power of organoids lies in their ability to be cultured from human patient-derived adult tissue-resident stem cells (ASCs) or pluripotent stem cells (PSCs), paving the way for personalised medicine and primary tissue disease modelling [18, 19]. Although, as with any model, organoids are not without limitations (See Text box: ‘Don’t believe the hype: organoid limitations’). Currently organoids have been used to investigate gene function, cell development, tissue and cellular level physiology and model host-microbiome interactions. They are also useful for modelling infectious and genetic diseases, investigating primary tumour growth, and have applications in drug screening and regenerative medicine [20, 21]. A further key driver of their utility, in addition to being a more physiologically relevant model, is their amenability to both standard and high-tech laboratory techniques and their genetic and molecular tractability. For example, organoids can easily be manipulated using viral and non-viral mediated delivery of CRISPR-Cas9 gene editing [22], they can be investigated using mass spectrometry [23], flow cytometry [24], multiple single cell ‘omics’ technologies [25] and naturally their 3D structure lends themselves to all manner of imaging technologies [26] (Fig. 1). This flexibility provides exciting opportunities for the generation of organoids from multiple organs and disease sources, coupled with their manipulation and phenotypic investigation.

## Mini-me: Modelling diabetes in a dish

**Fig. 2:**
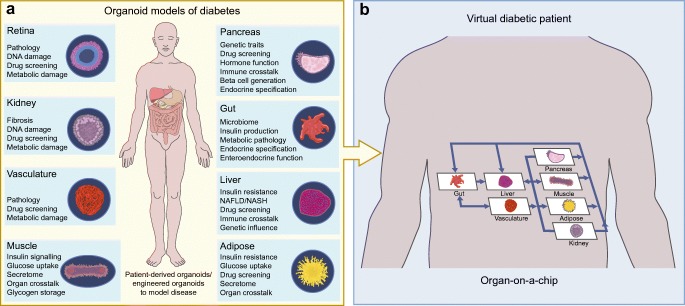
Mini-me: modelling diabetes in 3D. Diabetes is a multi-organ disease and organoids present an opportunity to generate models that more closely recapitulate its pathology. (**a**) Organoids can be generated from multiple tissues to allow the modelling of disease progression, investigation of genetic associations, screening of drugs and probing of mechanisms. (**b**) In the future, combining organoid technology and bioengineering may make it possible to model inter-organ communication in diabetes pathogenesis, creating a virtual diabetic patient on a chip. The image in (**b**) is adapted from [107], with permission from Elsevier. This figure is available as part of a downloadable slideset

Organoid technology has the power to accelerate diabetes research, particularly cell replacement therapies. The technology perfectly lends itself to the generation of novel sources of beta cells or the production of new cell-based delivery systems for insulin. These opportunities and the current state of the field are well described elsewhere [40–42]. There are, however, exciting opportunities beyond cell-based treatments of insulin-dependent diabetes. Organoids allow the modelling of primary disease tissues, insulin-sensitive tissues and peripheral tissues associated with diabetic complication, from primary sources (Fig. 2) without the need for transformed cells or large numbers of donors. We now have the tools to generate organoids from individuals with identified diabetes-associated genetic variants or to introduce these traits with CRISPR/Cas9 and use these organoids to provide a more precise evaluation of their contribution to diabetes pathogenesis. Results from these types of experiment may be informative in stratifying patients for particular interventions. These opportunities suggest that organoids may eclipse current in vitro models, transformed cell lines and short-term culture of primary tissues and provide a new understanding of diabetes pathophysiology.

## Tissues controlling glucose homeostasis

### Pancreas

The first pancreatic organoids were generated from mouse and human embryonic pancreatic cells by Anne Grapin-Botton’s group [43, 44]. These organoids produced progenitor-biased hollow spheres that could be differentiated into branched structures containing acinar, ductal and endocrine lineages. Pancreatic organoids have also been generated from cells derived from the adult pancreas; CD133^+^ cells isolated from the mouse pancreas can be expanded in vitro and differentiated towards all pancreatic lineages [45, 46]. However, organoids derived from human CD133^+^ cells require transgenic overexpression of *NGN3, MAFA and PDX1* to produce endocrine lineages [47].

Generation of islet organoids by turning 2D-directed differentiation of PSCs into 3D structures, has met with mixed success. Introduction of suspension cultures and an air–liquid interface produced immature beta cells that did not secrete insulin [48, 49]. In contrast, 3D islet-like organoids generated from either human embryonic stem cells [50] or from spontaneously formed endocrine cell clusters produced by stepwise differentiation of human PSCs [51] released insulin in response to glucose in vitro and in vivo. More recent efforts have introduced synthetic hydrogels [52] or co-cultures with HUVECs and mesenchymal stem cells (MSCs) combined with a self-condensation system [53]. These more advanced systems produced islet organoids with increased complexity and maturity, including endothelial cells and vascularisation.

Islet organoids provide potential improvements over the traditional pancreatic beta cell lines as they better mimic islet architecture and morphology. However, their apparent immaturity remains their major drawback. Islet organoids often do not recapitulate nutrient-stimulated insulin secretion adequately. As such, the current technology is not ready for prime-time functional exploration, leaving primary islets as the gold standard tool for assessing islet hormone secretion.

Pancreatic organoids are currently better suited to understanding pancreatic morphogenesis and differentiation. For example, a functional genetic screen in organoids derived from SOX9^+^ progenitors identified *Prdm16* as a novel regulator of islet development [54]. Pancreatic organoids could also provide a platform for drug screening and personalised medicine. PSC-derived organoids have been used to model pancreatic facets of cystic fibrosis and to screen a set of cystic fibrosis transmembrane conductance regulator (CFTR) activators [34]. It is also easy to envisage how organoids could help deepen our understanding of the processes that lead to immune destruction of beta cells or provide more precise details as to how genetic susceptibility loci may interact with the immune system to drive disease initiation and progression. Modelling these immune interactions could range from the simple addition of cytokines or conditioned media from cultured immune cells [55], to the co-culture of sorted and activated immune cells [56], or to more complex techniques developed for mimicking tumour immune microenvironments [57]. To date, few of these types of experiment have been leveraged for diabetes research but the potential is self-evident.

### Gut

The gut is a key player in the integration of luminal signals and the control of metabolism. It houses enteroendocrine cells (EECs) that produce over 20 different bioactive peptides implicated in the local control of absorption and motility, the signalling of satiety and augmentation of beta cell function [58, 59].

Organoids provide new opportunities to explore EEC differentiation, how this might be altered in diabetes and obesity or by dietary nutrients and how it could be targeted to manipulate the density of specific cell types for treating metabolic and other diseases.

The limited availability of donor tissue for islet transplantations has initiated an interest in identifying alternative sources. Several studies have explored using the gut as a potential source of insulin-producing cells for the treatment of diabetes. Both PSC- and ASC- derived organoids have been used to examine how EECs can be converted into insulin-secreting cells [60, 61].

Intestinal organoids also allow the investigation of the basic physiology of the gut epithelium and the function of EECs under physiological or metabolic disease conditions*.* For example, organoids have been used to investigate nutrient sensing, transport and absorption, lipid transport, hormone secretion and intracellular signalling processes [62]. The organoid platform will also help the microbiome field explore mechanisms of action. Microbial diversity is reduced in obesity, but we lack an understanding of the pathological implications. Protocols that give access to the apical (luminal) side of the organoid allow the controlled investigation of the microbiome and its metabolites [31, 39]. As yet, the generation of organoids derived from obese and/or diabetic individuals or those pre- and post-bariatric surgery, for example, have not been leveraged, but the platform offers a unique opportunity to ask critical questions of the role of the gut in the pathogenesis of metabolic disease and in the identification of mechanisms of metabolic surgeries or interventions. Finally, intestinal organoids may provide a finer understanding of the metabolic impact of nutrients on epithelial function at single cell and tissue resolution.

### Liver

The liver plays a central role in glucose homeostasis. Insulin resistance in the liver directly leads to hyperglycaemia and is also implicated in the pathogenesis of non-alcoholic fatty liver disease (NAFLD) and the subsequent development of non-alcoholic steatohepatitis (NASH). Current animal models of NAFLD and NASH do not perfectly mimic natural disease progression. Human liver organoids could provide a flexible tool for modelling the development of hepatic insulin resistance, studying glucose metabolism and hormonal responsiveness in the liver and identifying the underlying mechanisms driving NAFLD and its progression to NASH.

The first human liver organoids were generated from human induced PSC (iPSC)-derived hepatocytes, co-cultured with MSCs and endothelial cells, embedded in Matrigel. These original structures consisted mainly of proliferating hepatoblasts [63, 64]. More recently protocols have emerged to generate organoids from either human adult bile duct-derived bipotent progenitor cells [65] or primary hepatocytes [66, 67], these approaches are reviewed in [68, 69]. Steps to model NAFLD and NASH are under way. Exposing liver organoids from multiple species to fatty acids causes lipid accumulation, demonstrating proof of principle that organoids can be used to model aspects of NAFLD [70]. More recently, a comprehensive human model of steatohepatitis has been described by Takanori Takebe. Using healthy and diseased iPSCs, multicellular human liver organoids were derived, which, when exposed to NEFA, exhibited lipid accumulation, inflammation and fibrosis in a successive manner—key features of human steatohepatitis. This phenotype could be reversed with farnesoid X receptor (FXR)-treatment [71]. It is hoped that such a platform could be used to understand the mechanisms behind the progression of NAFLD to NASH and identify novel treatments for NAFLD/NASH, which currently have no approved pharmacological options.

### Muscle

The use of 3D primary tissue-derived cultures to model skeletal muscle in vitro pre-dates the modern organoid era. Vandenburgh et al. pioneered the development of bioartificial muscles (BAMs) generated by suspending myoblasts (muscle progenitor cells), isolated from muscle biopsies, in collagen/Matrigel and then casting them in a silicone mould containing two end attachment sites [72–74]. Following differentiation, parallel arrays of myofibres aligned in the direction of the attachment points and contracted when stimulated, but their size was limited because of the lack of a vasculature. This was addressed using a co-culture system containing HUVECs, which produced organoids consisting of aligned fibres with an integrated endothelial network [75]. However, human myoblasts have a limited expansion potential and an unstable differentiated state. To overcome these issues, several protocols have emerged for PSC-derived muscle organoids (reviewed in [76]). These organoids develop myobundles, exhibit contractility following electrical stimulation and can be engineered to include a vasculature and a nervous system [77].

These cultures have the potential to model insulin resistance in 3D but have only been studied in 2D. Iovino et al. generated myotubes from healthy volunteers and individuals with Donohue syndrome, a genetic disorder associated with mutations in the insulin receptor [78]. The myotubes derived from individuals with Donohue syndrome exhibited defects in insulin signalling, glucose uptake and glycogen accumulation, as well as insulin-regulated gene expression. In the future the challenge will be to model insulin resistance in human skeletal muscle organoids generated from diabetic primary or human PSC-derived myoblasts to better understand the development of insulin resistance and its consequences.

### Adipose tissue

Adipose tissue is a prominent site of insulin resistance in type 2 diabetic patients and is associated with increased chronic inflammation. Development of a human in vitro model to study the pathogenesis of adipose tissue in metabolic disease would be advantageous. There have been various attempts to generate 3D adipose cultures. Early protocols co-cultured human adipose stromal cells with HUVECS or used lipoaspirates and embedded them in either silk scaffolds or hydrogels [79, 80]. Differentiation of these cultures allowed lipid accumulation and these cultures secreted leptin, the archetypal adipokine. Other groups used adipose progenitors derived from the stromal-vascular fraction of human white adipose tissue and self-organised them into spheroids in hanging drops [81] or by first stirring and then embedding them in Matrigel they generated self-organised vascularised organoids [82]. These novel protocols open the door to using long-term patient-derived adipose cultures to explore the pathology of adipose tissue in metabolic disease.

## Modelling diabetic complications

Many of the long-term complications associated with diabetes are caused by microvascular damage, which leads to nephropathy, retinopathy and diabetic neuropathy. A paucity of accurate models that mimic the functional and molecular pathology of these complications has hampered our understanding of the disease mechanism involved and how the complications could be managed or prevented. Organoids may offer an opportunity to address this.

Exposure to hyperglycaemia causes abnormal thickening of the basement membrane of the vasculature, impairing the delivery of oxygen and nutrients to tissues, causing inflammation and damage. Generation of iPSC-derived human blood vessel organoids recapitulates the abnormal thickening of the basement membrane when they are exposed to hyperglycaemia. A subsequent drug screen using this model has identified a novel pathway for drug targeting, underscoring the potential of organoid disease modelling [83].

Human and mouse PSC-derived kidney organoids have been generated using a number of different protocols [84–86]. However, these protocols either failed to recapitulate the necessary cell types or produced disconnected nephrons and collecting ducts. Using optimised stepwise differentiation to separately generate nephron and ureteric bud progenitors before mixing in culture with embryo-derived stromal cells produced more refined kidney organoids [87]. In depth reviews of the protocols used for generation of kidney organoids and their uses are available [88–91]. Kidney organoids have yet to be leveraged for investigating diabetic nephropathy but there are several groups working in this area. The European Commission and the National Centre for the Replacement, Refinement and Reduction of Animals in Research (NC3Rs) have awarded funding for projects to establish in vitro models of diabetic nephropathy and kidney damage using PSC-derived organoids [92, 93]. The Diabetic Complications Consortium have also funded a project to model kidney fibrosis [94]. It will be exciting to see the outcomes of these innovative projects.

The human retina is a complex organ with no regenerative capacity, making it particularly sensitive to damage. Retinal organoids can be generated from both mouse and human ESCs and human iPSCs, which, when embedded in Matrigel, spontaneously form hemispherical epithelial optic vesicle, that invaginate and form the optic cup [95–97]. Further protocol refinement has enabled the generation of 3D retinal cups containing mature photoreceptors, an outer-segment-disc and demonstrable photosensitivity [98]. In depth information about retinal organoids is well described in other reviews [99–101]. Patient-derived retinal organoids offer an opportunity to more precisely understand the pathophysiology of diabetic retinopathy provide a platform for drug screening and will enable the exploration of genomic variants that render some diabetic patients more susceptible to retinal damage.

## The future in 3D

The arguments for applying organoid technology to diabetes research are persuasive. This technology has facilitated the generation of high-fidelity models of virtually any tissue in the body. They offer unprecedented predictive power over traditional 2D models, promise to bring speed and reliability to drug discovery and enable the discovery of novel disease mechanisms. However, organs do not exist in isolation; inter-organ crosstalk is highly relevant to pathophysiology, particularly for diseases like diabetes, which affect multiple organ systems. As such, organoids cannot replace whole body studies, but the field of bioengineering may provide opportunities to move towards a virtual diabetic individual. Organ-on-a-chip technologies have rapidly developed in a short space of time [102]. The technology allows the simultaneous culture of cells from different organs on a microfluidic chip, allowing the precise control of flow between compartments, nutrient supply, shear stress and local mechanical and electrical properties [103]. These systems have often relied on 2D cultures; the challenge now will be to combine organ-on-a-chip technology and 3D organoid technology. Human islet organoids derived from human iPSCs have been generated on an organ-on-a-chip platform [104]. PSCs were initially differentiated into embryonic bodies followed by endoderm differentiation, islet differentiation and maturation. Theses organoids contained heterogeneous islet-like components and functionalities and may resemble their native tissue more closely than static organoid cultures. There are several commercial companies who have developed specialised organ-on-a-chip equipment, ranging from simple multi-well plate systems using gravity to drive flow [105], to complete chip-based pump perfused technologies [106]. It is easy to envisage a future where we will be able to link key human organoid tissue models using chip-based technology to investigate organ-level communication in the pathogenesis of diabetes. Organoid technology is poised to enable researchers to transform our understanding and treatment of diabetes.

## Electronic supplementary material

Slideset of figures (PPTX 642 kb)

## Abbreviations

ASC: Adult tissue-resident stem cell
EEC: Enteroendocrine cell
HIO: Human intestinal organoid
iPSC: Induced pluripotent stem cell
MSC: Mesenchymal stem cell
NAFLD: Non-alcoholic fatty liver disease
NASH: Non-alcoholic steatohepatitis
PSC: Pluripotent stem cell
