# Supplementary material for: Diabetes through a 3D lens: organoid models
Source: Diabetologia. 2020 Mar 27;63(6):1093–102. doi: 10.1007/s00125-020-05126-3 (PMC7228904; doi:10.1007/s00125-020-05126-3)
Supplement: Supplementary file 1 — (PPTX 642 kb) [file 125_2020_5126_MOESM1_ESM.pptx]

## Slide 1
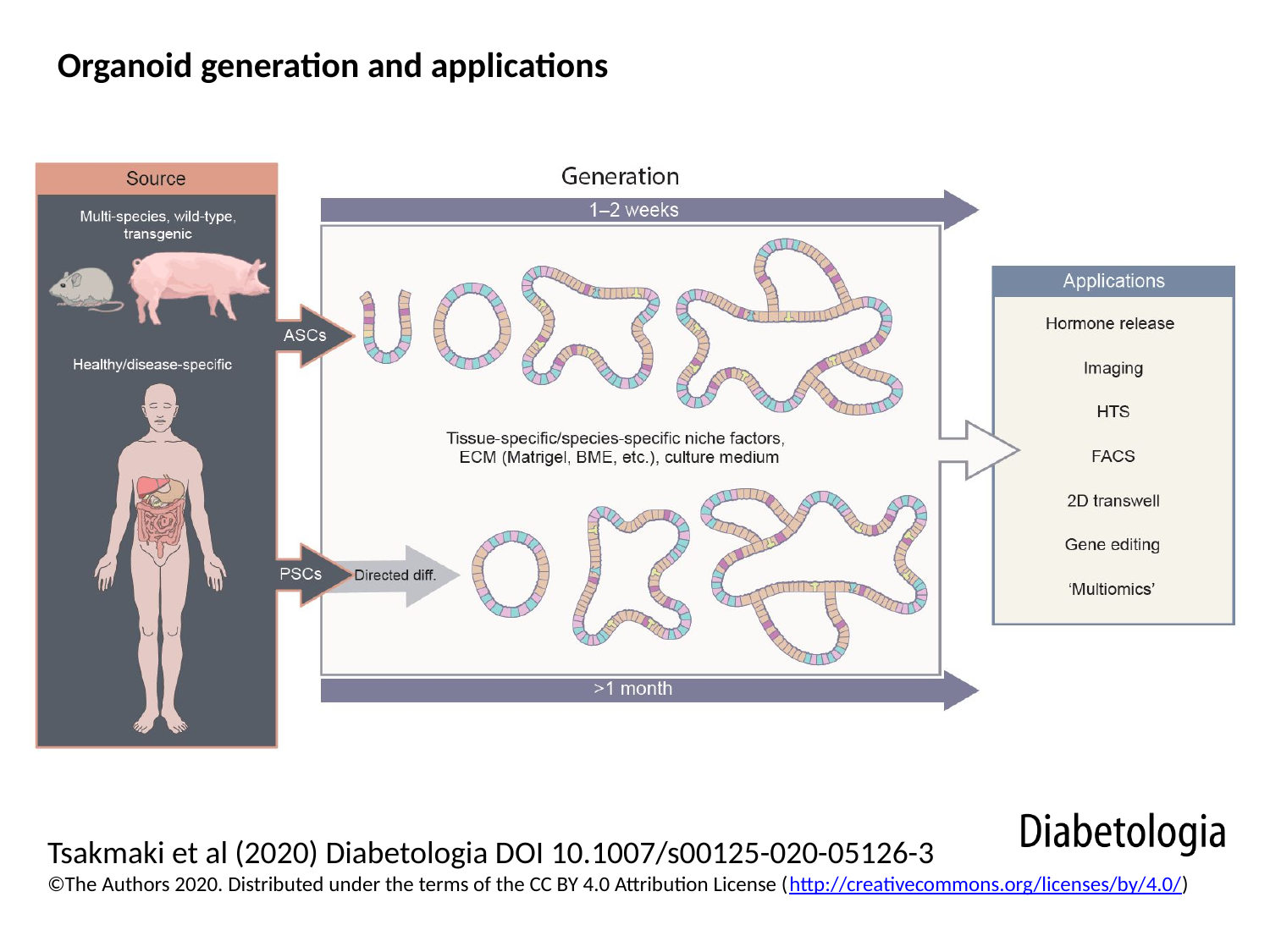

Organoid generation and applications
Tsakmaki et al (2020) Diabetologia DOI 10.1007/s00125-020-05126-3
©The Authors 2020. Distributed under the terms of the CC BY 4.0 Attribution License (http://creativecommons.org/licenses/by/4.0/)

## Slide 2
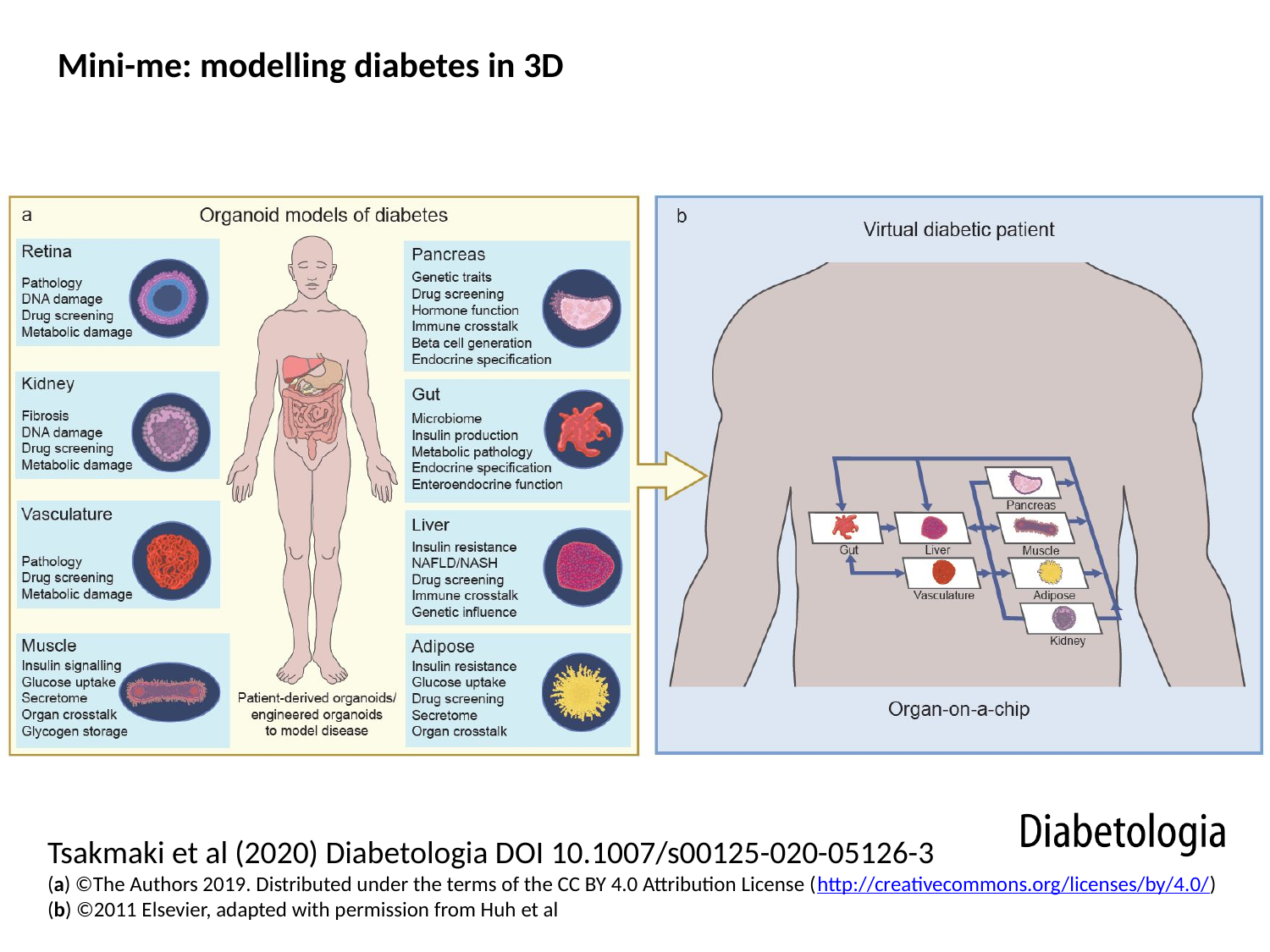

Mini-me: modelling diabetes in 3D
Tsakmaki et al (2020) Diabetologia DOI 10.1007/s00125-020-05126-3
(a) ©The Authors 2019. Distributed under the terms of the CC BY 4.0 Attribution License (http://creativecommons.org/licenses/by/4.0/)
(b) ©2011 Elsevier, adapted with permission from Huh et al
